# Follicular Thyroid Carcinoma Relapse Cases - Revisited by X-ray 3D Virtual Histology

**DOI:** 10.1007/s12022-025-09891-y

**Published:** 2025-11-24

**Authors:** Kiarash Tajbakhsh, Olga Stanowska, Jonas Bossart, Marija Buljan, Antonia Neels, Martina T. Mogl, Catarina Alisa Kunze, Guenther Klein, Wolfgang Hulla, Rene Brillmann, Sabine Kirchnawy, Michael Hermann, Reto Kaderli, Robert Zboray, Aurel Perren

**Affiliations:** 1 Center for X-ray Analytics, Swiss Federal Laboratories for Materials Science and Technology (Empa), Dubendorf, 8600 Switzerland; 2 Department of Chemistry, University of Fribourg, Fribourg, 1700 Switzerland; 3 Institute of Tissue Medicine and Pathology, University of Bern, Bern, 3008 Switzerland; 4 Nanomaterials in Health, Swiss Federal Laboratories for Materials Science and Technology (Empa), St. Gallen, 9014 Switzerland; 5 Swiss Institute of Bioinformatics (SIB), Lausanne, Switzerland; 6 Department of Health Sciences and Technology, Eidgenössische Technische Hochschule Zürich (ETH), Zürich, Switzerland; 7 Charité - Universitätsmedizin Berlin, Corporate Member of Freie Universität Berlin and Humboldt-Universität zu Berlin, Berlin, Germany; 8 Institute of Pathology, Charité - Universitätsmedizin Berlin, Corporate Member of Freie Universität Berlin and Humboldt-Universität zu Berlin, 10117 Berlin, Germany; 9 Landesklinikum Wiener Neustadt, Vienna, Austria; 10 Jakob-Erdheim Institute of Pathology and Clinical Bacteriology, Wiener Gesundheitsverbund Klinik Hietzing, Vienna, Austria; 11 Klinik Landstrasse, Department of Surgery, Wiener Gesundheitsverbund, Vienna, Austria; 12 Department of Visceral Surgery and Medicine, Bern University Hospital, Inselspital, University of Bern, Bern, Switzerland; 13 ARTORG Center, University of Bern, Bern, 3010 Switzerland

**Keywords:** Follicular thyroid neoplasms, Digital pathology, X-ray 3D virtual histology, Micro-CT

## Abstract

The diagnosis and prognosis of follicular thyroid neoplasms are based on the identification of capsular and vascular invasion. Although conventional histology allows accurate classification in most cases, its inherent limitations in tissue sampling can result in misjudgment of both the presence and extent of invasion, and occasionally result in diagnostic inaccuracies. Consequently, unexpected tumor recurrence or overtreatment still represent significant clinical challenges. To mitigate these limitations, emerging diagnostic approaches are exploring advanced imaging modalities. X-ray 3D virtual histology has been reported to enable non-destructive and comprehensive sampling of the entire tumor volume embedded in formalin-fixed paraffin-embedded blocks. In thisstudy, the X-ray 3D virtual histology technique is first evaluated by classifying 99 follicular thyroid carcinomas and 31 follicular adenomas, achieving an accuracy of 89.2%. It is then applied to tissue blocks from five relapse cases that were postoperatively diagnosed as adenomas using conventional histology. Three of five tumors exhibited at least one unequivocal focus of vascular invasion, reinforcing that even a single well-defined focus portends significant risk of recurrence and distant metastasis. Although histological confirmation at this stage remained necessary, X-ray 3D virtual histology proved to be a valuable screening method.

**Supplementary Information:**

The online version contains supplementary material available at 10.1007/s12022-025-09891-y.

## Introduction

Follicular thyroid neoplasms represent 5 to 15% of the thyroid tumors [1]. The treatment strongly depends on the diagnostic outcome and includes surgical therapy, and in case of malignancy, complementary radioiodine therapy. Follicular adenomas (FA) are non-invasive, encapsulated follicular thyroid neoplasms without cytological and architectural features of papillary thyroid carcinoma. The malignant counterpart, follicular thyroid carcinoma (FTC), is distinguished by capsular invasion (CI) and vascular invasion (VI). Their distinction in both preoperative ultrasound and intraoperative frozen section examination is straightforward when it comes to widely invasive FTCs. However, it remains challenging in recognizing encapsulated angioinvasive and minimally invasive FTC which require diagnostic histopathological examination of the whole tumor capsule [1–3].

Minimally invasive FTC is characterized by the presence of minimal CI and an excellent prognosis. However, the standalone prognostic value of CI remains under debate, and future classification systems may revisit its significance. If VI is observed in a tumor with an intact capsule, then it is classified as encapsulated angioinvasive. Oncocytic thyroid carcinoma (OTC) previously recognized as a histological variant of FTC, is now recognized as a separate entity, yet shares the same subclassification and paradigm: minimally invasive, encapsulated angioinvasive, and widely invasive.

VI is a well-established adverse prognostic factor, strongly associated with distant metastases and reduced overall survival [4]. According to the current World Health Organization (WHO) guidelines, both the presence and extent of VI are prognostically relevant. The number of foci of VI has to be documented as the tumors with extensive angioinvasion (> 4 foci) have worse prognosis [1]. Extensive angioinvasion has been associated with significantly worse prognosis, with a study reporting an odds ratio of 13.7 for 10-year cause-specific mortality [5]. This aligns with reported differences in disease-free survival rates at 40 months: 97% for minimally invasive FTC, 81% for encapsulated angioinvasive FTC, and 45% for widely invasive FTC [6]. The 2025 American Thyroid Association (ATA) guidelines classify FTC and OTC with extensive VI as high-risk with greater than 30% risk of recurrence.

The 2025 ATA guidelines upstages the risk of recurrence for FTC and OTC with limited angioinvasion (< 4) from low-risk (2–3%) to intermediate risk (10–30%) based on studies suggesting that any VI is associated with more aggressive behavior [4, 7]. A study applying rigid diagnostic criteria, in which intravascular tumor was diagnosed only when associated with fibrin or clear invasion of the vessel wall, reported a higher risk of distant metastasis [8]. Under these rigid criteria, the number of foci of VI did not influence the outcome [8].

In cases where exhaustive histological evaluation reveals ambiguous capsular or vascular features, the 2022 WHO classification introduces a diagnostic category termed “tumor of uncertain malignant potential” (UMP) [1]. These tumors are defined as well-differentiated, encapsulated or well-circumscribed follicular-patterned neoplasms in which invasiveness cannot be confidently established (ruled out or confirmed) despite thorough sampling and examination.

Complete inspection of the tumor capsule is therefore essential for accurate prognosis and treatment planning of FTC, which remains a significant challenge in endocrine histopathology. While the conventional guideline suggests embedding at least one formalin-fixed paraffin-embedded (FFPE) block per centimeter of tumor diameter, a more rigorous approach recommends embedding two FFPE blocks per centimeter to ensure comprehensive tumor capsule embedding [9]. Current slide preparation protocols typically recommend preparing only one histological slide per FFPE block. Given the thickness of individual histological Sects. (2–10 μm), only a small fraction of tumor capsule is examined per slide, leaving much of the 3D capsule volume unsampled. Deeper sections may be prepared if initial slides suggest but do not confirm invasive behavior, yet full step sectioning of all the relevant blocks is not performed routinely. Such an approach would require the generation and review of hundreds of slides per case, which is impractical in routine workflows.

Processing artifacts also contribute to diagnostic challenges. These may include mechanical tissue distortion, excessive heating during the dewaxing process, overdrying before staining, or prolonged exposure to certain fixative chemicals [10]. Last but not least, CI may be mimicked by post–fine needle aspiration (FNA) changes, further complicating the diagnostic assessment [11]. Consequently, both underdiagnosis and overdiagnosis can occur, impacting therapeutic decision-making and patient outcomes.

Recent advances in imaging technologies now enable high-resolution, three-dimensional visualization of soft tissues, and opening new avenues for digital pathology [12]. One such advancement is X-ray 3D virtual histology (X3DVH), recently applied to the evaluation of follicular thyroid neoplasms [13, 14]. This non-destructive technique enables direct imaging of FFPE blocks without additional preparation, readily fitting into clinical workflows. At an isotropic resolution of ~ 20 μm, meaning the voxel size is equal in all three spatial dimensions, a single block can be scanned within about one hour, making it feasible for routine batch imaging (exact durations are provided in the Supplementary Materials). As a true 3D modality, it is particularly well-suited for applications requiring comprehensive spatial assessment [12, 15], such as the evaluation of CI and VI in follicular thyroid neoplasms.

To assess the potential of X3DVH for FTC diagnosis, a collaborative initiative was launched in partnership with the German Association of Endocrine Surgeons (CAEK). In the first step, the method was assessed on a cohort of 99 FTC and 31 FA FFPE tissue blocks, with gross classifications (malignant vs. benign) compared against histological ground truth. In the second step, FFPE tissue blocks from five thyroid tumors initially diagnosed as FA but later relapsed were inspected with X3DVH. These cases were obtained from tissue banks from the university hospitals in Vienna, Berlin, Zürich, and Bern. In total, five relapse cases were examined in the second part of the study.

## Materials and Methods

### Micro-CT

Micro-CT was performed using a commercial EasyTom XL Ultra scanner (Rx-Solutions, Chavanod, France), equipped with a Hamamatsu L10801 reflection target microfocus X-ray source and a high-resolution flat panel detector. The detector includes a high-efficiency CsI scintillator, a 127 μm pixel pitch, a 1880 × 1494 pixel array, and a 16-bit dynamic range.

Scans were conducted between February 2023 and January 2025, with parameters adjusted based on sample geometry, availability of scan time, and evolving technical considerations. For the relapse cases, 36 FFPE tissue blocks with capsule embeddings were imaged in total. Scans were typically performed on paired FFPE blocks arranged horizontally, and the reported parameters reflect this dual-block setup [16]. Following whole-block scanning, regions of interest were identified by K.T., and targeted high-resolution scans were performed on selected subregions.

The details of scan parameters are provided as an Excel sheet in the supplementary materials. The median value of the whole block scan parameters were as follows: tube voltage = 60 kV, tube current = 350 µA, tube power = 24 W, frame rate = 8.25 fps, 50-frame averaging per projection, and 1440 projections per scan. Each acquisition lasted approximately 2 h 40 min. The source-to-object and source-to-detector distances were 52.24 mm and 412.28 mm, respectively, resulting in a magnification factor of 7.98 and an effective voxel size of 15.93 μm. For the validation cohort, the scan parameters were within the same range, and the above values are representative.

The system was also equipped with an alternative detector: a 14-bit CCD featuring a 9 μm pixel size. This detector was typically operated in 2 × 2 binning mode, as its 20 μm-thick Gadox scintillator limited the achievable physical resolution to approximately 20 μm. While less efficient than the flat panel detector due to the thinner scintillator layer, it is better suited for high-resolution applications and propagation-based phase-contrast imaging. This detector was used exclusively for the local scans, which were optimized for edge-enhancement [16].

Image reconstruction was performed using the Feldkamp-Davis-Kress algorithm with filtered back projection [17], yielding isotropic voxel resolution. For local high-resolution scans, a single-distance phase retrieval step was applied using a variable Paganin filter cut-off frequency at −6 dB [18], as specified in the Excel sheet in supplementary materials.

## Case Selection

### Relapse Cases

FFPE blocks of primary and recurrent thyroid tumors of 9 patients from 7 institutions, with or without corresponding Hematoxylin and Eosin (H&E) stained slides, were obtained from collaborating parties affiliated with the CAEK, and the archives of the Institute of Tissue Medicine and Pathology (ITMP). In cases lacking histological slides, the FFPE blocks were sectioned at 2 μm thickness using a Leica HM325 rotary microtome. The resulting sections were mounted and stained with H&E using a Tissue-Tek Prisma automated tissue processor.

All slides were reviewed by experienced endocrine pathologists (O.S. and A.P.). All available (see Table 1) FFPE blocks from primaries confirmed as non-invasive follicular thyroid neoplasms without papillary carcinoma features and paired with their recurrences (local or in form of distant metastases, see Table 2) were qualified for the study if at least 4 FFPE blocks of at least 3 mm thickness containing tumor periphery were present.

**Table 1:** Confusion matrix of X3DVH performance

	Predicted Positive	Predicted Negative	Total
Actual Positive	TP = 85	FN = 14	99
Actual Negative	FP = 0	TN = 31	31
Total	85	45	130

**Table 2:** Clinical summary of relapse cases included in the study. The table lists preoperative diagnosis (Pre-Op Dx), year of surgery, original pathological diagnosis, year of relapse, and relapse site for each case (R1–R5)

Case	Pre-Op Dx	Operation	Original pathological diagnosis	Relapse	Relapse site
R1	Big tumor with irregular border on USG, Tg > 1000	2010	Oncocytic FA	2019	Local relapse as encapsulated angioinvasive OTC
R2	Scintigraphic cold tumor by goiter, no FNA	2017	Oncocytic FA	2024, 2025	Local relapse, lung metastasis
R3	Symptomatic tumor, no FNA	2016	FA	2024	Bone metastasis
R4	Scintigraphic cold tumor by goiter, no FNA	2012	Oncocytic FA	2022	Lung metastases
R5	Bethesda IV by FNA	2011	FA	2023	Local relapse as widely invasive OTC

*Tg * thyroglobulin level (ng/mL), *USG* ultrasonography

### Validation Cohort

For validation of X3DVH, we analyzed an ITMP archive cohort of 130 diagnostic FFPE tissue blocks comprising 99 histologically confirmed FTCs and 31 FAs. In case of FTCs, the blocks selected for X3DVH comprised histological features of invasion.

### X3DVH

#### Case Studies

All FFPE blocks containing tumor capsule from the selected cases were provided to K.T. for X3DVH analysis. The 3D reconstructions were assessed in a blind manner by K.T., without access to the corresponding H&E slides. K.T. annotated the volumes for VI and CI, and those annotations were reviewed by experienced pathologist O.S. In cases where consensus was reached, the locations were marked for targeted step-sectioning, and the suspected features were confirmed or refuted by histological examination.

#### Validation Cohort

X3DVH scans were independently reviewed by K.T., who annotated regions suspicious for CI and VI. These annotations were subsequently discussed with an expert pathologist (O.S.) to review suspected invasive features. No subsequent histological confirmation was conducted for the invasion sites deep in the block. The histological diagnosis therefore served as ground truth against which the diagnostic (malignant vs. benign) performance of X3DVH was assessed.

#### Step Sectioning

Additional step-sectioning was performed at 30 μm intervals using a MICROM HM 355 S microtome (Thermo Scientific) based on annotations provided by K.T. and verified by O.S. on X3DVH. These sections underwent H&E staining and whole-slide imaging. Slides suggestive of VI underwent further evaluation using Elastic-Van Gieson (EVG) staining to visualize elastic fibers within vessel walls and clarify vascular structures.

## Results

### X3DVH Validation

The resulting classification performance is summarized in Table 1. This reflects the gross classification of each FFPE block as malignant (FTC) or benign (FA), rather than the detection of individual invasive features or subclassification of FTC. X3DVH achieved a sensitivity of 85.9%, specificity of 100%, accuracy of 89.2%, positive predictive value (PPV) of 100%, and negative predictive value (NPV) of 68.9%.

These findings demonstrate that X3DVH exhibits high specificity. Practically, this implies that when a block is classified as malignant by X3DVH, it is almost certainly malignant, consistent with the PPV observed in this cohort. However, the method failed to identify 14 blocks as FTC that were confirmed by histological analysis, resulting in reduced sensitivity and a lower NPV. Therefore, a benign classification by X3DVH cannot reliably exclude malignancy, and histological confirmation remains essential to establish benign status. Furthermore, even in blocks accurately classified as malignant, histology remains indispensable for confirmation of foci of invasion. The reduced sensitivity of X3DVH is attributable to its lower resolution and contrast relative to conventional histology, reflecting a trade-off for the acquisition of volumetric data. Given the method’s high specificity, its performance in cases misclassified by conventional histology warrants investigation, as the added volumetric information may offer diagnostic utility.

### Case Studies

Following the validation analysis, X3DVH was applied to five challenging cases in which conventional histology had led to misclassification. These comprised five tumors initially diagnosed as FA but later relapsed (R1–R5), with details of the initial diagnosis and relapse site summarized in Table 2. This cohort provided an opportunity to test X3DVH in clinically relevant scenarios where volumetric information could complement standard histology.

Table 3 provides an overview of the case study results. Cases are categorized according to the presence or absence of VI, while CI was not considered for classification because of its lesser importance and perfect prognosis. In three cases (R1, R2, and R5), X3DVH successfully identified at least one VI, which was subsequently confirmed by targeted serial sectioning. In R4, the diagnosis was revised to UMP, while R3 no additional invasive features were detected. R3 is undersampled in terms of tumor capsule embeddings with 4 blocks prepared for a 6 cm diameter tumor. Thus, invasive features could have been missed during tumor capsule embedding. Except for R3, X3DVH contributed to revising or refining the initial diagnosis of cases that had been misclassified as adenomas.

**Table 3:** Summary of cases assessed by X3DVH and histology. The number of VI sites is listed under the columns “X3DVH” highlighting features detected by 3D virtual histology and under “Histology,” the ones subsequently confirmed or refuted by targeted serial sectioning. The table reports the number of FFPE blocks containing tumor capsule, indicating the extent of tissue sampling relative to tumor diameter, with the number scanned by X3DVH shown in parentheses

Case	X3DVH	Histology	Tumor diameter (cm)	FFPE blocks with tumor capsule (#scanned)	Tumor/parenchyma interface entirely submitted
R1	3	1	7.3	9 (9)	yes
R2	0	1	4	5 (5)	no
R3	1	0	6	4 (4)	no
R4	1	*	3.5	7 (7)	yes
R5	1	1	6.5	12 (11)	yes

* Capsular discontinuity with vascular structures and inflammation: indeterminate etiology—tumor invasion versus FNA artifact.

### R1

**Fig. 1:**
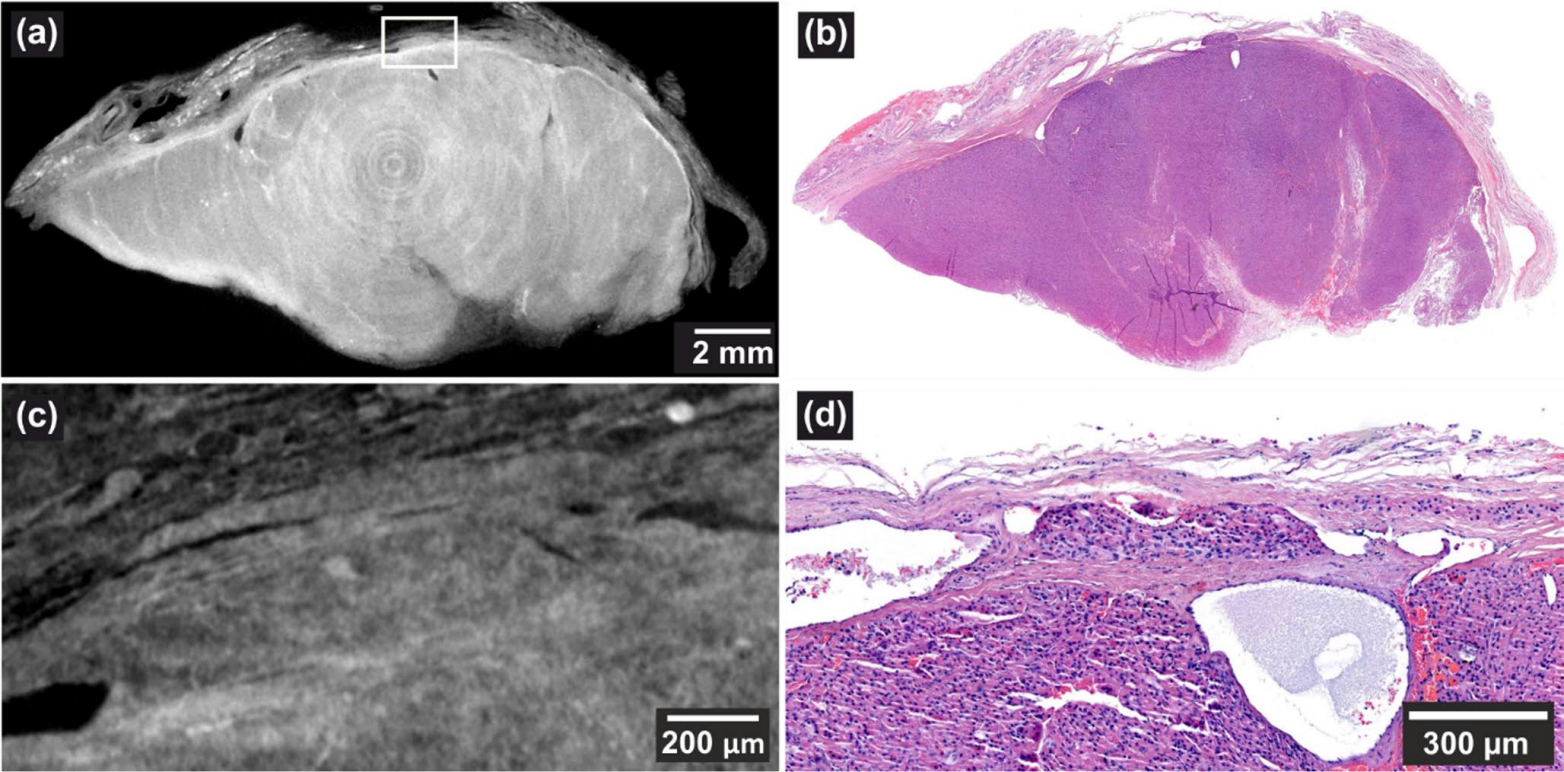
(**a**, voxel size: 13.90 μm) VI detected ~ 800 μm deep in the FFPE block using X3DVH. (**b**) Corresponding H&E section shown. (**c**), voxel size: 3.00 μm) A high-resolution local scan, and (**d**), depth: ~1360 μm) matching high-magnification H&E image confirm the finding

This case concerns a 66-year-old male who underwent thyroidectomy in 2010. In 2019, the patient experienced rise in Tg level of 2500 ng/mL and retrosternal nodule was found, consistent with encapsulated angioinvasive OTC. This relapse indicates that the original lesion must have harbored malignant features that went unrecognized at the time of the initial diagnosis. Following histological reexamination of nine FFPE blocks from the primary tumor capsule, which showed no suspicious features, the specimen was subsequently submitted for X3DVH screening according to the study protocol. Three regions within a single block were identified as suspicious for VI, with one confirmed as a definite VI. As illustrated in Fig. 1, the VI was located approximately 800 μm deep in the block and was initially detected through a whole-block scan (voxel size = 13.90 μm), shown in (a). A high-resolution local scan at a 3.00 μm voxel size shown in (c), provided enhanced visualization, while high-magnification H&E in (d) confirmed the presence of VI. Several dilated vessels were also observed in the vicinity, further supporting the diagnosis.

Of the remaining two suspicious invasion foci, one was identified as a septum, while the other was located at the edge of the tissue and was partially cut off during sectioning. According to WHO classification guidelines, these findings would lead to a revised diagnosis of encapsulated angioinvasive FTC.

### R2

This case involves a 69-year-old female who was diagnosed with right-sided oncocytic FA in 2017. In late 2023, right-sided cervical nodule was detected, and FNA in March 2024 revealed tumor cells consistent with thyroid oncocytic follicular neoplasm. A right lateral neck dissection was subsequently carried out in April 2024, followed by lung segment resection of metastatic focus, again histologically confirmed as oncocytic differentiated carcinoma, consistent with thyroid carcinoma metastasis.

**Fig. 2:**
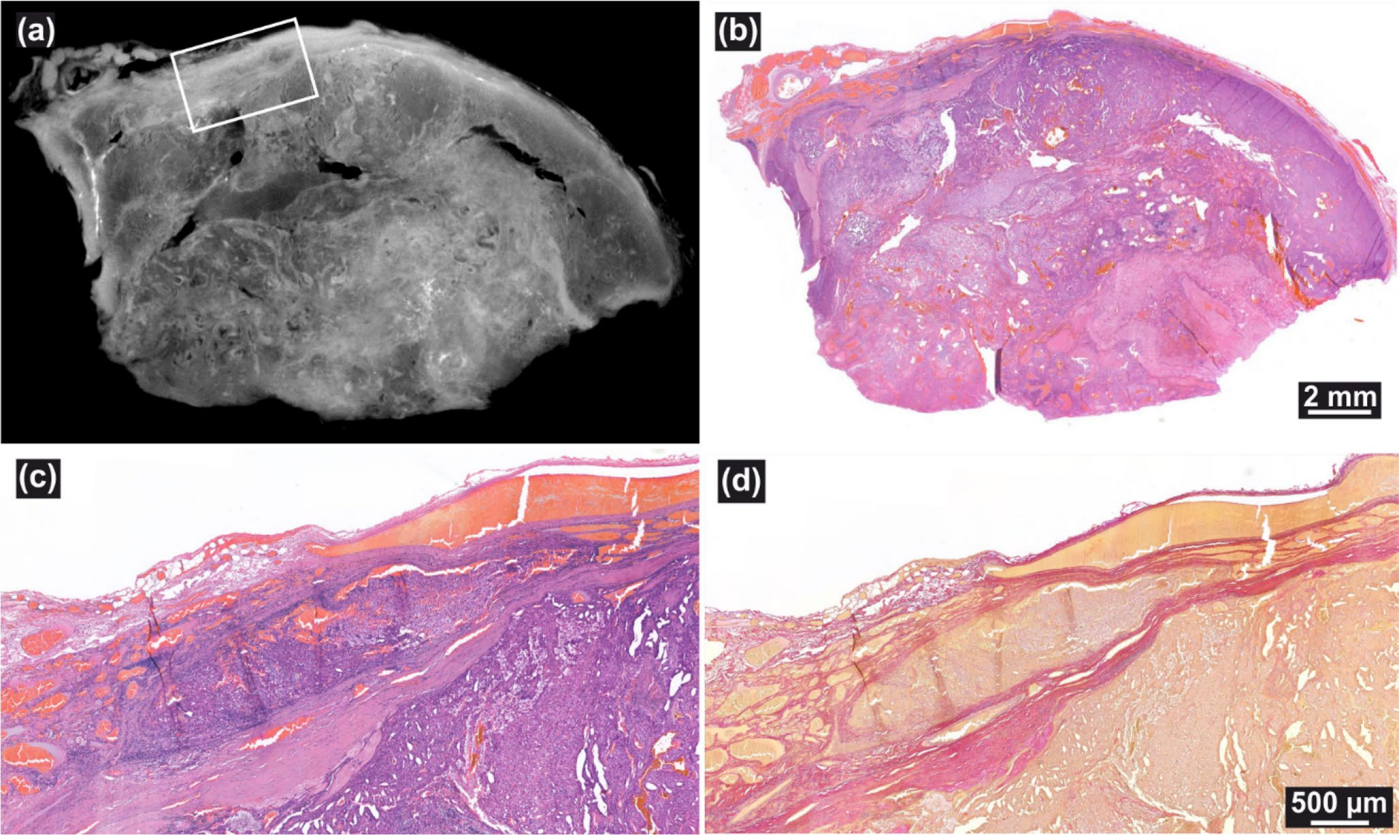
(**a**, voxel size: 12.7 μm) VI identified ~ 800 μm deep in the FFPE block, and (**b**) the corresponding H&E section at the same depth, with high-magnification detail in (**c**) and the corresponding EVG stain in (**d**)

For the study purpose, five FFPE blocks from the primary 4 cm tumor were examined by X3DVH. While the topmost slice of the FFPE block appeared non-invasive, deeper levels revealed a capsular irregularity measuring approximately 4 mm in diameter, which was located approximately 800 μm deep in the tissue (Fig. 2(a)). However, no clear VI pattern was initially identified. Given that this was the only morphological abnormality seen, targeted serial sectioning was performed. Deeper sections confirmed the presence of VI, as illustrated in Fig. 2(c) H&E and (d) EVG images.

### R3

This case concerns a 42-year-old female who underwent right-sided hemithyroidectomy in June 2016. Notably, for a 6 cm dominant tumor within a goiter, only four blocks were sampled from the tumor periphery, suggesting the absence of grossly suspicious features. In 2024, the patient presented with a bone lesion, and a core biopsy revealed a Tg-positive follicular neoplasia, suggestive of metastatic thyroid-origin disease. A subsequent complementing hemithyroidectomy of the left lobe in August 2024 also resulted in a diagnosis of FA. Although no definitive malignant features were identified in either thyroid lobe, and no ovarian mass was present as a potential alternative source of a primary (i.e., malignant struma ovarii), the presence of distant metastases raises the possibility that carcinoma may have been missed in the 6 cm thyroid nodule.

**Fig. 3:**
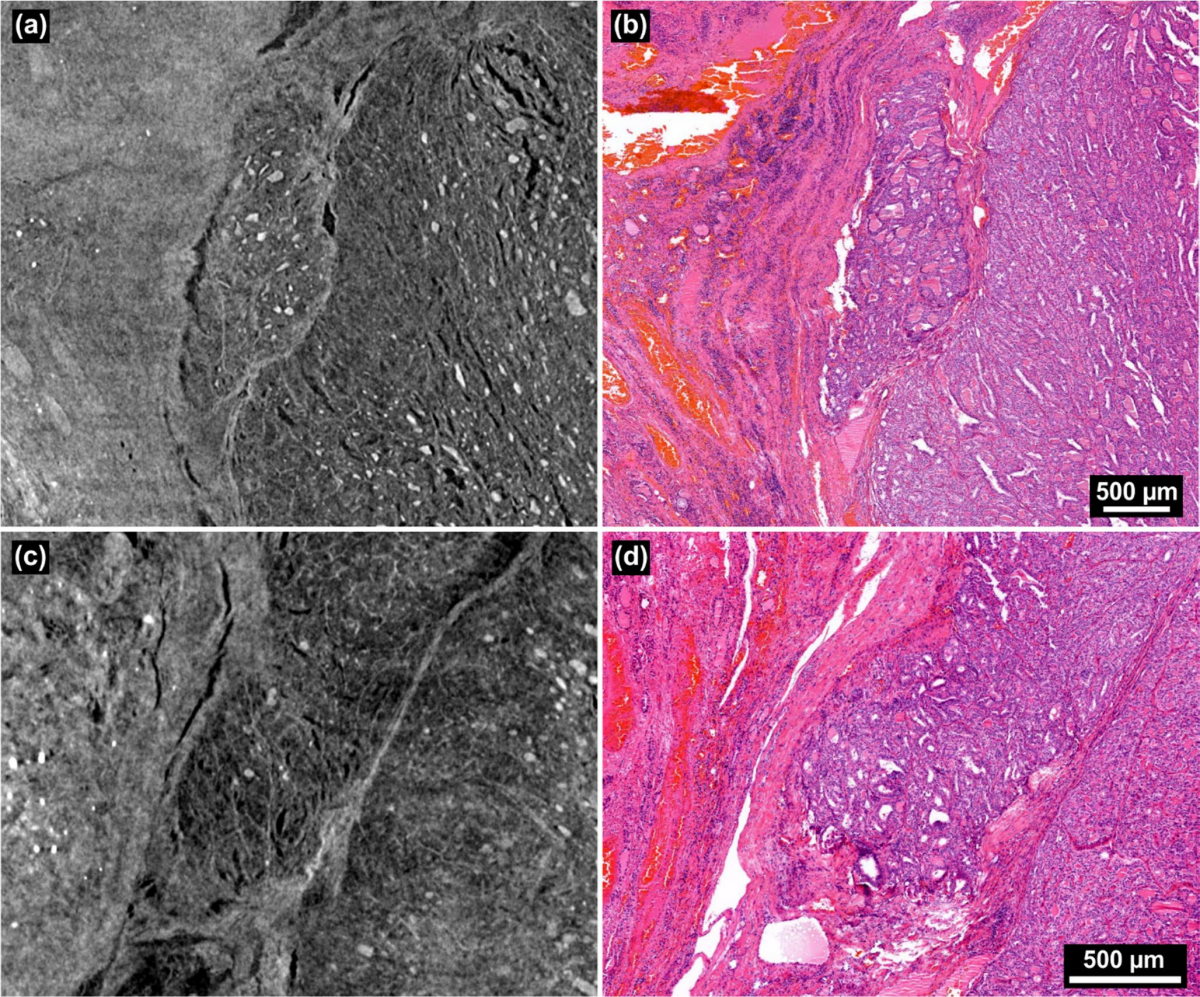
(**a**, voxel size: 4.6 μm) A suspected VI by X3DVH, which proved to be a false positive by histological inspection in (**b**). The second suspected invasion is shown in (**c**), voxel size: 4.9 μm), and its corresponding H&E image in (**d**), also negative for invasion

Upon histological review, no suspicious areas of invasion were identified. X3DVH analysis of the initially resected lobe identified two adjacent regions, approximately 3.5 mm apart that were suspicious for VI (see Fig. 3(a) and (c)). However, serial histological sectioning and review determined both foci to be borderline or equivocal, potentially intracapsular, but lacking definitive evidence of vascular wall penetration (Fig. 3(b) and (d)). Notably, these ambiguous regions were located near the FFPE block cutting plane, an area prone to mechanical deformation and artifact-induced variability. Additionally, the presence of inflammation and inward capsule deflection toward the tumor center raises concerns about a possible histologic alteration following fine-needle aspiration of the thyroid. This phenomenon is encompassed by the term WHAFFT (Worrisome Histologic Alterations Following Fine-Needle Aspiration of the Thyroid), which refers to a spectrum of reactive changes such as pseudoinvasion of the tumor capsule and infarct-like necrosis that can mimic malignancy in thyroid tissue following FNA [19].

### R4

This case concerns a 54-year-old female patient diagnosed with FA in 2012. A decade later, in 2022, the patient experienced a relapse, with Tg levels rising above 300 ng/mL and small pulmonary foci detected, raising suspicion for metastatic disease. On October 13, 2022, three locally recurrent nodules were surgically resected.

**Fig. 4:**
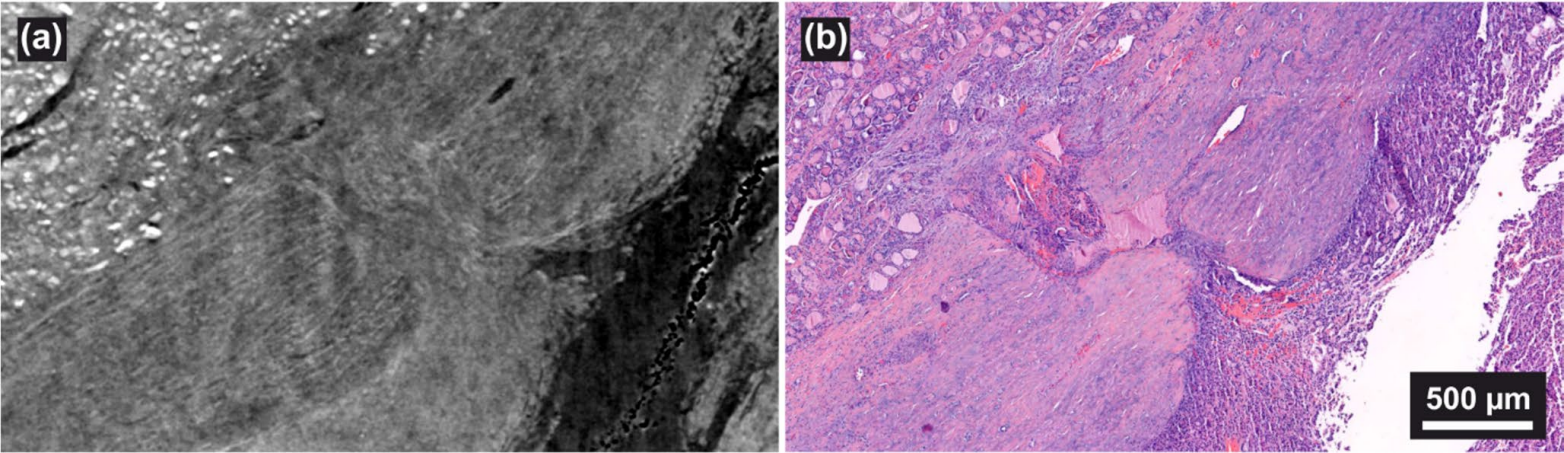
(**a**, voxel size: 3.7 μm) The capsule irregularity was observed in X3DVH image at approximately 500 μm deep in the blocks, its corresponding H&E image is shown in (**b**)

No suspicious areas of invasion were observed on histological re-examination. X3DVH screening was conducted on seven FFPE blocks of the primary tumor. One block revealed a narrow capsular interruption approximately 500 μm below the surface, raising concern for VI, as shown in Fig. 4(a). Targeted serial sectioning and.

corresponding H&E staining of this region confirmed the same focal discontinuity of the capsule (Fig. 4(b)). The capsule appeared split with a rare morphology that may be confounded by surrounding inflammation. Although suspicious, this alteration lacked definitive features of VI and CI. Based on these findings, the lesion was classified as a UMP. The atypical capsular configuration may reflect WHAFFT.

### R5

This case involves a 67-year-old male who was diagnosed with FA in 2011 after hemithyroidectomy. In 2023, he experienced a relapse, with the recurrent lesion diagnosed as a widely invasive OTC measuring 14.5 cm, confirmed to have VI sites and staged as pT3. The stark contrast between the initial benign diagnosis and the aggressive recurrence highlights the potential for histologically indolent-appearing lesions to undergo malignant progression over time.

**Fig. 5:**
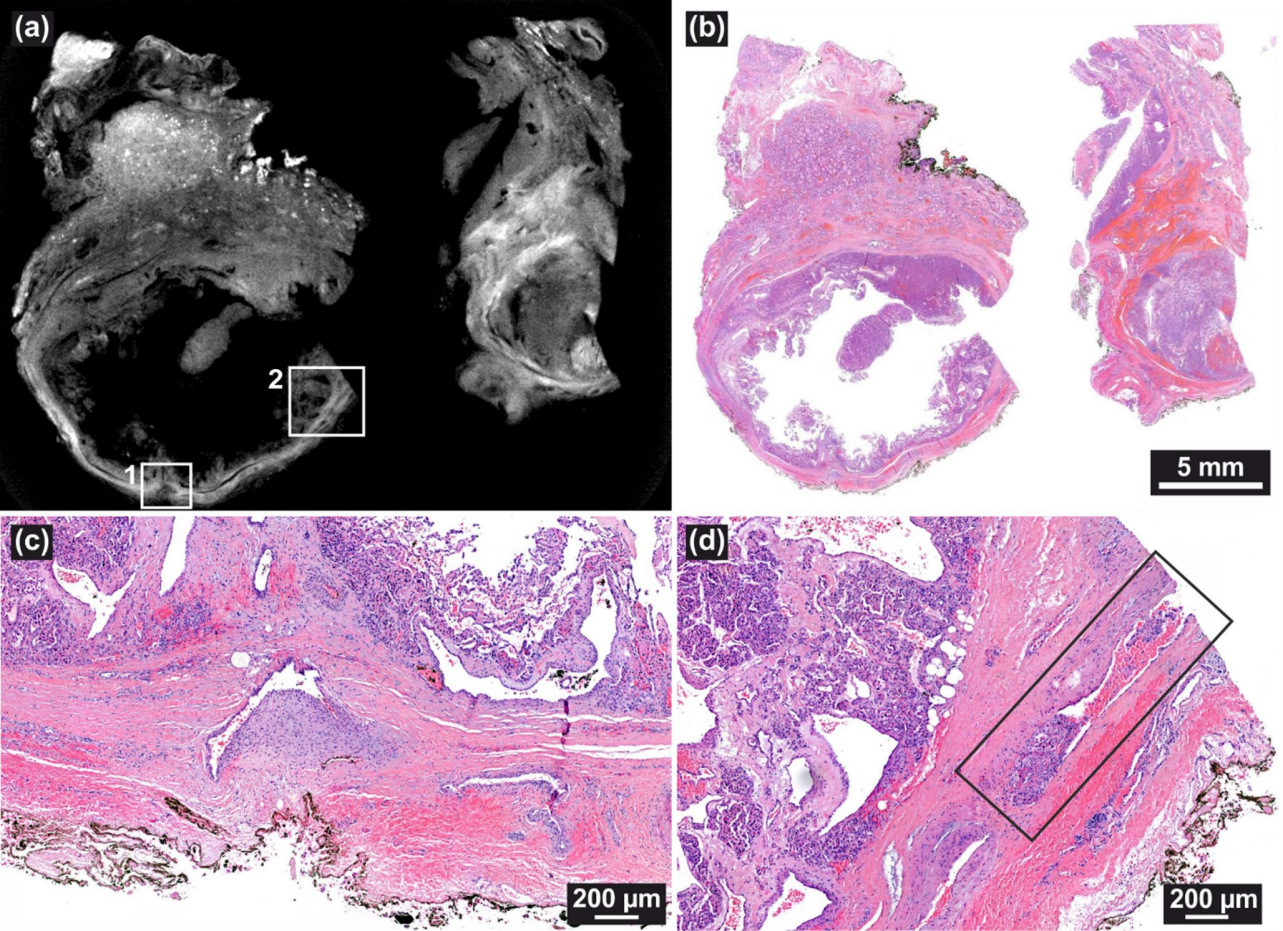
(**a**, voxel size: 22.3 μm) X3DVH scan showing a suspicious but histologically refuted VI (ROI 1) and a VI that was discovered by serial sectioning in (ROI 2). The corresponding H&E section at ~ 450 μm depth is shown in (**b**). ROI 1 was discarded histologically (**c**), while ROI 2 confirmed a true VI (**d**)

Nine FFPE blocks encompassing the tumor-capsule interface were examined. Histological revision revealed no evidence of invasive foci. X3DVH promptly identified two discrete foci of CI in separate blocks. However, histological assessment of the primary tumor was complicated by artifacts from mechanical sectioning, including tumor fragmentation and contour distortion, particularly due to the presence of a thin, hemorrhagic capsule. Therefore, the suspected features were discarded as artifacts. In a second block, X3DVH suggested a possible VI presented in Fig. 5(a) region of interest (ROI 1). While serial sections revealed that this putative VI was artifactual, resulting from an irregularly thickened vessel wall (Fig. 5(c)). Through serial sectioning multiple small caliber VI sites were revealed that could not be resolved by X3DVH (Fig. 5(a) ROI 2).

The histological image is shown in Fig. 5(e). Histologically identified VI in Fig. 5(b) was discovered in the vicinity of ROI 1 which is shown in high magnification in Fig. 5(e). The presence of multiple VIs clarifies the biological basis of the patient’s tumor relapse. This VI should be in the resolution limit of X3DVH, but because of the short scan time dedicated to the sample, it was missed.

## Discussion

This study is retrospective in nature, which inherently introduces the potential for bias in the evaluation and selection of tissue samples. This limitation should be considered when assessing the performance and generalizability of the proposed imaging methodology.

None of the reference adenomas in the validation cohort exhibited the borderline morphological features identified in our index cases. This finding reinforces the notion that malignancy in follicular thyroid tumors does not always manifest through unequivocal CI or VI, as classically defined. Instead, these tumors lie along a continuum of morphological alterations, with borderline and ambiguous features that challenge the binary classification of benign versus malignant. Such gray-zone cases expose the limitations of current histopathological criteria and underscore the need for more refined diagnostic tools for follicular thyroid neoplasms. Every classification will be a compromise between risk of recurrence and overtreatment.

In our cohort, two patients with angioinvasive thyroid carcinoma that progressed to distant metastasis exhibited a single focus of VI. These findings suggest that applying rigid diagnostic criteria of VI appears to be more important than counting the number of invaded vessels, as even a single true focus of VI was associated with metastatic spreads [8].

To achieve an accurate diagnosis of these neoplasms, sufficient sampling of the tumor capsule is essential. However, it is a labor- and resource-intensive endeavor. The main advantage of X3DVH compared to conventional histology is the ability to efficiently capture the entire 3D tumor volume within a block, rather than relying on selected thin sections. This reduces the risk of missing invasion due to limited sampling and avoids alterations associated with slide preparation, even if embedding-related mechanical artifacts and sampling bias remain. It allows following the course of the vessel and picture the actual event of tumor cells invading through a vessel wall which allows to differentiate from pseudo-invasion that is the biggest pitfall of two-dimensional histology.

X3DVH images, characterized by lower contrast and limited cellular details, differ markedly from the high-contrast, cell-rich appearance of conventional H&E-stained sections. Consequently, they may initially seem unfamiliar or challenging to interpret. Nonetheless, the pathologists participating in this study adapted rapidly to the grayscale morphological features presented by X3DVH, indicating that broader adoption is feasible with appropriate training. The enhanced spatial context and volumetric data offered by X3DVH have the potential to serve as a valuable adjunct to traditional histology, particularly once pathologists become familiar with its distinct visual language.

To further support interpretation, techniques such as false-color rendering could be considered [20], but we argue that maintaining the native grayscale format preserves the fidelity of the original information. Artificial color mapping, particularly in low-contrast settings, risks introducing misleading artifacts and should be used cautiously. Instead, a more complementary strategy lies in the integration of artificial intelligence. Deep learning models can be trained to detect relevant morphological features and generate attention maps that highlight suspicious regions within the volume. These attention maps can serve as guides for pathologists, streamlining their review process and reducing the cognitive load of navigating complex 3D data. Such a synergistic approach offers a practical and scalable path toward clinical translation.

In line with this vision, radiomics and deep learning-based image analysis have already demonstrated their potential as alternatives to conventional tissue characterization by extracting quantitative features from medical images. For example, micro-CT was used in combination with deep-learning and spatial transcriptomics to provide a 3D spatial transcriptomics landscape [21, 22]. Micro-CT deep-learning has also shown superior performance to 2D histology in prostate cancer prognostication [23]. Recent studies, suggest that radiomics models trained on micro-CT data can predict mutation status and spatially map molecular alterations such as *TERT* or *BRAF* mutations [24]. These imaging advances with deep-learning models, offer new opportunities for non-invasive, spatially resolved tumor profiling.

The ability of X3DVH continues to improve in terms of contrast and resolution. Brighter X-ray sources [25], more efficient detectors with lower readout noise [26], and smaller pixel sizes will enhance image quality [16]. Taken together, these developments indicate that X3DVH holds potential for clinical translation in the evaluation of thyroid tumors.

## Conclusion

Undersampling of the tumor capsule remains a conundrum, often originating at the grossing stage due to macroscopically inconspicuous nodules, such as those with a thin capsule or occurring in the context of multinodular goiter. This limitation may result in the preparation of fewer FFPE blocks than necessary, potentially compromising diagnostic accuracy. Sectioning 4 μm-thick tissue slides from 4 mm-thick FFPE blocks further limits representative spatial sampling. However, with the advent of X3DVH technology, imaging of the prepared blocks, which previously required full serial sectioning, can now be completed non-destructively in approximately one hour. This enables subsequent targeted step-sectioning to confirm tumor invasion. In the validation cohort, X3DVH achieved a sensitivity of 85.9%, specificity of 100%, accuracy of 89.2%, PPV of 100%, and NPV of 68.9%, confirming its high specificity but also its lower sensitivity in detecting all invasive features compared to histology. In 3 out of 5 relapsed cases examined, X3DVH revealed diagnostically important features. However, it failed to detect suspicious features in two cases, in one of which the number of available FFPE blocks was insufficient relative to tumor size. In summary, X3DVH offers a screening method for the initial inspection of the tumor volume and capsule, facilitating targeted step-sectioning prior to histological analysis. Nevertheless, due to its lower sensitivity, it cannot yet be considered a standalone diagnostic tool at this stage.

## Supplementary Information

Below is the link to the electronic supplementary material.

Supplementary Material 1(XLSX 15.9 KB)

## Data Availability

No datasets were generated or analysed during the current study.
